# Disparate age and sex distribution of sessile serrated lesions and conventional adenomas in an outpatient colonoscopy population–implications for colorectal cancer screening?

**DOI:** 10.1007/s00384-022-04191-x

**Published:** 2022-06-04

**Authors:** Vidit Lall, Ali Galalah Mostafa Ismail, Oyekoya Taiwo Ayonrinde

**Affiliations:** 1 Sir Charles Gairdner Hospital, Nedlands, Australia; 2 Royal Perth Hospital, Perth, Australia; 3 Medical School, The University of Western Australia, Nedlands, Australia; 4 Department of Gastroenterology and Hepatology, Fiona Stanley Hospital, Murdoch, Australia; 5 Faculty of Health Sciences, Curtin University, Bentley, Australia

**Keywords:** Colorectal cancer (CRC), Colonoscopy, Sessile serrated lesion (SSL), Conventional adenoma, Risk factors, Young-onset colorectal cancer

## Abstract

**Purpose:**

Colorectal cancer (CRC) is increasingly diagnosed in individuals aged < 50 years, resulting in advocacy of screening from age 45 years. Despite existing knowledge associating CRC with conventional adenomas, the significance of sessile serrated lesions (SSLs) on the burden of CRC is less detailed. We aimed to provide contemporary estimates for SSL prevalence and examine patient and procedure factors associated with SSL detection.

**Methods:**

Retrospective observational study examining associations between SSL and conventional adenoma detection, polyp histopathology, patient, and procedure characteristics in an outpatient colonoscopy unit over 12 months.

**Results:**

From 2097 colonoscopies, SSL detection was 13.8% overall and 12.5% in patients < 50 years. SSLs were mostly proximal in location (64%), and SSL detection was significantly higher in females compared with males (16.2% vs. 11.7%, *p* = 0.003), particularly in those < 50 years (16.8% vs. 8.6%, *p* < 0.001). In multivariable analysis, SSL detection was associated with female sex (adjusted odds ratio [aOR] 1.48, 95% confidence interval [CI] 1.15–1.91), synchronous conventional adenoma detection (aOR 1.36, 95% CI 1.04–1.78) and BMI ≥ 25 kg/m^2^ (aOR 1.34, 95% CI 1.02–1.77). Conventional adenoma detection was 33.6% and associated with age ≥ 50 years (aOR 3.57, 95% CI 2.84–4.47) and synchronous SSL detection (aOR 1.36, 95% CI 1.03–1.79).

**Conclusions:**

We observed age and sex disparities in polyp types and prevalence in this outpatient colonoscopy population. SSLs were most prevalent in females aged < 50 years, suggesting a potential increased susceptibility of young females to SSLs and CRC. Our findings may have implications for the design of CRC screening programs.

## Introduction

CRC is the third most common cancer worldwide and a second-leading cause of cancer deaths. The burden of CRC is particularly high in Australia, ranking among the top five regions for CRC incidence [1]. Risk factors include polyps, diverticular disease, and a low-fibre diet [2]. As CRC rates continue to rise globally, ongoing efforts are being made to better understand the changing epidemiology, risk factors, prevention, screening, and treatment of CRC. Rising CRC rates in people < 50 years of age is increasingly being observed [3].

Approximately two-thirds of CRCs are derived from conventional adenomas, henceforth referred to as adenomas. However, the serrated neoplasia pathway is another important contributor to CRC, accounting for approximately one-third of sporadic CRCs [4–6]. The World Health Organisation (WHO) currently identifies three different types of serrated polyps: hyperplastic polyps (HP), sessile serrated lesions (SSL−previously known as sessile serrated adenomas/polyps), and traditional serrated adenomas (TSA) [7]. SSLs are of contemporary interest, as their detection may be challenging and awareness regarding their prevalence and malignant potential is increasing [4, 5]. Furthermore, SSLs seem to be associated with a disproportionately high number of interval CRCs compared to their reported prevalence [8]. This has raised questions about whether current screening paradigms for CRC appropriately target SSL detection.

Histopathologically, the WHO recently defined SSLs as “serrated polyps with ≥ 1 unambiguous distorted crypt”, which is more sensitive than the previous diagnostic criteria from 2010 requiring “pathological features in two or three adjacent crypts” [7, 9]. This has led to a considerable proportion of previously diagnosed HPs being reclassified as SSLs [5, 10]. Molecularly, the serrated pathway is known to exhibit high amounts of cytosine-phosphate-guanine (CpG) island methylation and can express microsatellite instability. This is also known as the CpG island methylator phenotype (CIMP) [11, 12]. The reported prevalence of SSLs varies from 1.1 to 15% depending on the study population and year examined, which may reflect the use of different diagnostic criteria and geographical variations. The variation in SSL detection is also observed among studies in the same geographic region and the similar time period and may reflect advancing knowledge, individual endoscopist and pathologist experience, and improvements in colonoscope technology.

There is also ongoing controversy regarding risk factors associated with SSLs, with high variability seen for both endoscopist SSL detection rate and histopathological identification [4, 13–17]. Factors contributing to these inconsistencies include (a) the flat morphology of SSLs and their frequent location in the proximal colon, (b) periodic updates of SSL histopathological diagnostic criteria by the WHO, most recently in 2019, and (c) the fact that SSLs rarely bleed, resulting in low detection utilising the faecal immunochemical test (FIT) as a sole screening tool [4, 15, 18]. In Australia, the current minimum SSL detection rate of 4%, set as a quality measure by the Gastroenterological Society of Australia (GESA) for colonoscopy recertification of endoscopists [19], appears somewhat arbitrary and is probably inadequate relative to emerging knowledge.

### Aims

We aimed to provide contemporary estimates for SSL prevalence and identify patient and procedure characteristics associated with SSL detection.

## Methods

### Study design

Our study was a retrospective observational study of an outpatient colonoscopy population at a non-tertiary hospital. Patient and procedure characteristics were recorded from the clinical records. Study approval was obtained from the East Metropolitan Health Service Governance, Evidence, Knowledge, Outcomes Committee (activity approval 32886) and did not require written or informed consent because of the low-risk, retrospective, and non-interventional design of the study.

### Study population and colonoscopy characteristics

All colonoscopies performed in an outpatient day surgery unit between Jan 1 and Dec 31, 2019, were included in the study (*n* = 2097). Patient data were obtained from referral letters and clinical records. Data recorded included age, sex, body mass index (BMI), and colonoscopy indication. Patients undergoing surveillance for inflammatory bowel disease (IBD) were not excluded. Patient characteristics that were not consistently documented, such as ethnicity, smoking status, and alcohol consumption, were not included. Colonoscopy data obtained included endoscopist speciality, quality of bowel preparation, withdrawal time, colonoscopy findings including the size and location of colorectal polyps, the presence of mass lesions, and incidental findings such as diverticular disease and haemorrhoids.

### Colorectal polyp histopathology data

Polyp histopathology reports were reviewed, and histologic characteristics including the presence of dysplasia were recorded. Polyp types of interest were SSLs, adenomas, HPs, and TSAs. Adenomas were subclassified as tubular, tubulovillous, or villous adenomas. Advanced adenomas were defined as adenomas with a size ≥ 10 mm, a prominent villous component, or high-grade dysplasia. Clinically significant serrated polyps (CSSPs) were defined as SSLs, TSAs, proximal colon HPs ≥ 5 mm, or HPs ≥ 10 mm anywhere in the colon [20]. Molecular pathology information was not available in histopathology reports. We defined proximal colorectal polyps as polyps in the caecum, ascending colon, hepatic flexure, and transverse colon, while distal colorectal polyps were at or distal to the splenic flexure (i.e. splenic flexure, descending, sigmoid colon, and rectum) as previously described [21]. Individual endoscopist adenoma detection rate (ADR) and SSL detection rates (SDRs) were calculated.

### Statistical analysis

Data are summarised as median (interquartile range), means (standard deviation), or proportions. Univariate analyses were performed to examine associations between the presence of SSLs or adenomas and individual patient and procedure characteristics using Pearson Chi-square tests or Student’s *t* tests. Missing data were omitted from analyses. All *p* values were reported as two-sided and were interpreted at the 5% level of significance. For correlations between SSL detection and adenoma detection, we included data on endoscopists who performed more than 100 colonoscopies during the study period. Multivariable logistic regression analyses were conducted, including patient demographic and colonoscopy-related characteristics that were significantly associated with the polyp types in univariate analyses. HPs were not included in multivariable predictive models for SSLs and adenomas, as they were not considered clinically significant. The outcome variables are SSL, CSSP, and adenoma detection. Odds ratios (OR) and 95% confidence intervals (CI) for SSL, CSSP, and adenoma detection are reported. OR is presented as adjusted OR (aOR) in multivariable analysis. All statistical analyses were performed with the Statistical Packages for the Social Sciences (SPSS), version 26 (IBM Corp., Armonk, NY, USA).

## Results

### Patient cohort factors

Amongst the 2097 colonoscopies performed in the study, 1365 (65%) involved polypectomy. The median patient age was 54 (41–63) years, and 46.6% of patients were female. The mean BMI was 26.5 (5.5) kg/m^2^. The mean age did not differ significantly, comparing males versus females (51.8 years for males, 52.0 years for females, *p* = 0.798), nor did mean BMI (26.4 kg/m^2^ for males, 26.6 kg/m^2^ for females, *p* = 0.406). Reasons for colonoscopy are summarised in Table 1. Some colonoscopies were performed for multiple indications.

**Table 1:** Colonoscopy indications divided into three categories

**Indication category**	**Blood loss indication**	**Non-bleeding GI symptoms**	**Screening/surveillance for polyps and CRC**
*Percentage of colonoscopies*	49.5%	38.6%	33.4%
	•Iron deficiency •Rectal bleeding •Positive occult blood •Anaemia	•Altered bowel habit •Weight loss •Bloating •Tenesmus •Abdominal pain	•Positive FIT •Personal history of CRC •Family history of CRC •Previous polyp detection

*GI* gastrointestinal, *CRC* colorectal cancer, *FIT* faecal immunochemical test

### Procedure factors

Colonoscopies were performed by specialist gastroenterologists (64.9%) or general surgeons (35.1%), with nine colonoscopists performing more than 100 colonoscopies each during the study year. Colonoscopes used were the Olympus 180 series. Bowel preparation was good (reported as excellent or adequate) in 93% of colonoscopies based on colonoscopist impression. A total of 29.8% of patients had diverticular disease. Chromoendoscopy use was documented in < 1% of colonoscopies. Withdrawal times were only reported in 50% of colonoscopies, however, were consistently longer than 6 min where documented.

### Polyp detection

Fifteen pathologists were involved in polyp diagnosis in our study. SSL detection was 13.8% overall, with most SSLs located in the proximal colon (64.4% proximal vs. 35.6% distal, *p* < 0.001) and having no dysplasia (99%). Approximately 30.4% of SSLs without dysplasia were > 10 mm. The percentage of SSLs located in the proximal colon was not significantly different between sexes or between patients under and over 50 years (*p* > 0.05). Only one patient had a formal diagnosis of serrated polyposis, as consistent with recent WHO guidelines for gastrointestinal neoplasia [7]. The distribution of polyps is shown in Table 2. Most adenomas were tubular adenomas with low-grade dysplasia (90.3%), followed by tubulovillous adenomas with low-grade dysplasia (8.1%). Six patients had adenocarcinoma (0.29% of all colonoscopies). Nine out of 2097 patients had incomplete polyp data.

**Table 2:** Detection rates of serrated lesions and conventional adenomas as a percentage of all colonoscopies

			**Serrated lesions**			**Conventional adenomas**	
	***SSLs***	***HPs***		***TSAs***	***CSSPs***	***Adenomas***	***AAs***
**% of colonoscopies**	13.8	26.4		0.8	17.6	33.6	9.8

*SSL* sessile serrated lesion, *HPs* hyperplastic polyps, *TSAs* traditional serrated adenomas, *CSSPs* clinically significant serrated polys, *adenomas* conventional adenomas, *AAs* advanced adenomas

### Univariate analysis

#### Associations between patient cohort factors and SSL detection

**Fig. 1:**
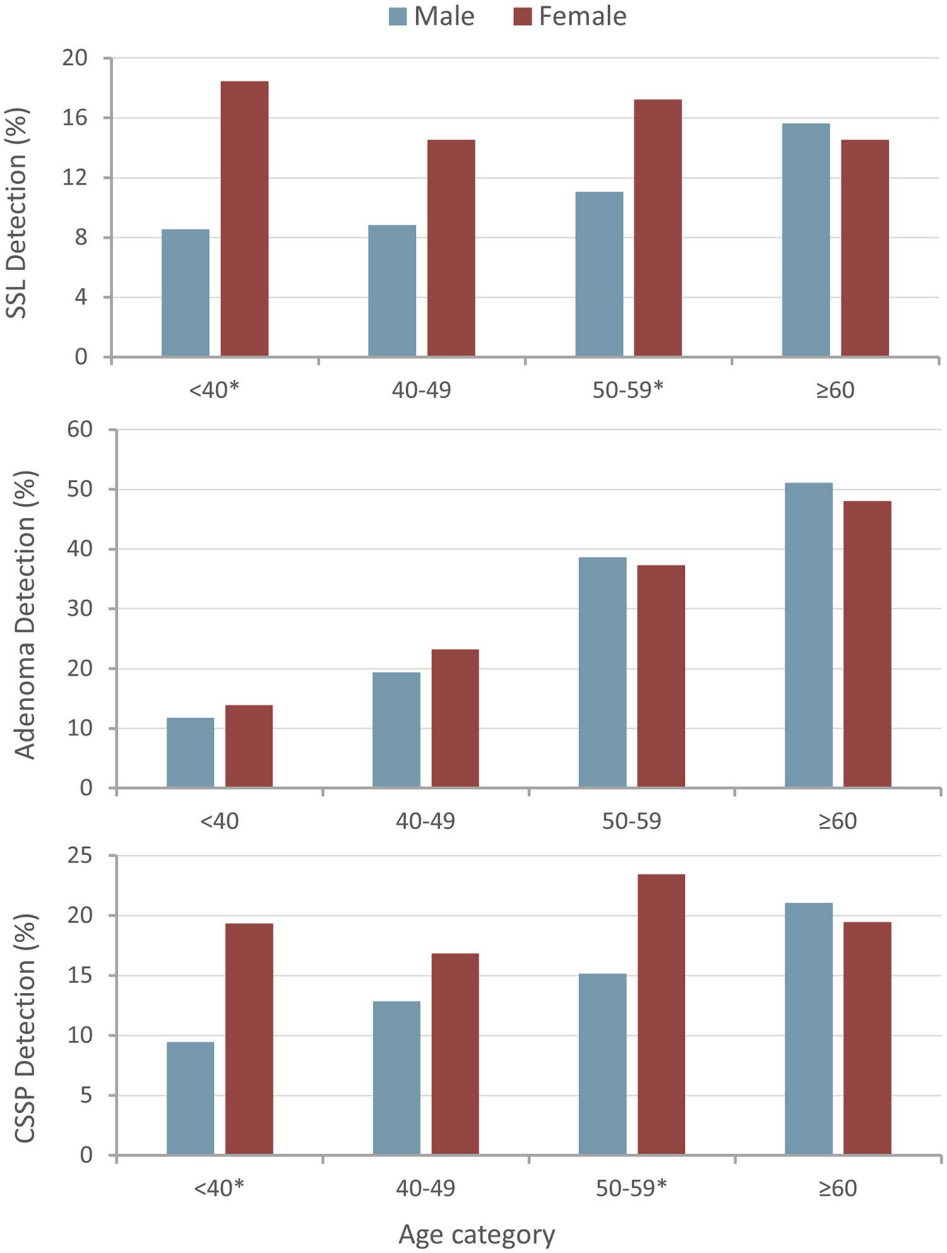
Detection rates of SSLs, conventional adenomas (adenomas), and CSSP (as % of all colonoscopies) by various age categories and sex. Age groups marked with an * represent those with significant differences between males and females (*p* < 0.05)

The associations between patient cohort factors and SSL detection are summarised in Table 3. SSL detection was not significantly different between patients < 50 and ≥ 50 (12.5% and 14.7%, respectively, *p* = 0.17). However, comparing males and females, SSL detection was higher in females than males (16.2% vs. 11.7% respectively, *p* = 0.003). This sex difference was most significant in patients aged under 50 years (females 16.8% vs. males 8.6%, *p* < 0.001). By contrast, there was no significant sex difference in SSL detection in patients aged above 50 years (15.8% vs. 13.7%, respectively, *p* = 0.67). The distribution of SSL detection by sex and age categories is shown in Fig. 1. Amongst younger patients aged < 40 years, SSL detection was significantly higher in females compared to males (18.4% vs. 8.5%, respectively, *p* = 0.002). Considering other patient characteristics, SSL detection was higher in patients with BMI ≥ 25.0 kg/m^2^ compared to BMI < 25.0 kg/m^2^ (15.2% vs. 11.3%, respectively, *p* = 0.02). A total of 17.8% of colonoscopies had a positive FIT status and of these, 18.0% included detection of an SSL. SSL detection was not predicted by colonoscopy indication nor a positive FIT (*p* = 0.10).

**Table 3:** Associations between individual patient cohort factors and SSL detection

**Patient cohort factor**		Total*	**SSLs**		***p*** **value**
			***Yes (n*=** ***288)***	***No*** **(n** **=** ***1800)***	
**Sex**					**0.003**
* Male*		1120 (53.4%)	**131 (11.7%)**	987 (88.3%)	
* Female*		977 (46.6%)	**157 (16.2%)**	813 (83.8%)	
**Age categories**					
< *40*					**0.002**
	*Male*	247 (53.2%)	**21 (8.5%)**	226 (91.5%)	
	*Female*	217 (46.8%)	**40 (18.4%)**	177 (81.6%)	
* 40–49*					0.10
	*Male*	181 (51.3%)	16 (8.8%)	165 (91.2%)	
	*Female*	172 (48.7%)	25 (14.5%)	147 (85.5%)	
* 50–59*					**0.04**
	*Male*	299 (55.6%)	**33 (11.0%)**	266 (89.0%)	
	*Female*	239 (44.4%)	**41 (17.2%)**	198 (82.8%)	
≥ *60*					0.67
	*Male*	391 (53.6%)	61 (15.6%)	330 (84.4%)	
	*Female*	339 (46.4%)	49 (14.5%)	290 (85.5%)	
**Colonoscopy indication**					0.10
*Blood loss*		1038 (49.5%)	152 (14.6%)	886 (85.4%)	
*Non-bleeding GI symptoms*		809 (38.6%)	97 (12.0%)	712 (88.0%)	
*Screening or surveillance*		700 (33.4%)	110 (15.7%)	590 (84.3%)	
**BMI**					**0.02**
< *25.0 kg/m*^*2*^		749 (36.0%)	**85 (11.3%)**	664 (88.7%)	
≥ *25.0 kg/m*^*2*^		1331 (64.0%)	**202 (15.2%)**	1129 (84.8%)	

*SSL* sessile serrated lesion, *GI* gastrointestinal, *BMI *body mass index

*Totals less than the overall number of colonoscopies (2097) occur where data is missing for the respective variables.

#### Associations between patient cohort factors and adenoma detection

Adenoma detection was significantly higher in patients aged ≥ 50 compared with < 50 years (45.0% vs. 16.8%, respectively, *p* < 0.001). This finding was consistent in males (45.7% vs. 15.6%, *p* < 0.001) and females (44.2% vs. 18.0%, *p* < 0.001). There was no significant difference in adenoma detection between sexes (males 33.9% vs. females 33.2%, *p* = 0.30). Adenoma detection was highest in patients with screening/surveillance as the only indication for colonoscopy (38.5%) and was significantly associated with a positive FIT (OR 1.96, 95% CI 1.40–2.73) and BMI ≥ 25.0 kg/m^2^ (OR 1.56, 95% CI 1.29–1.90).

#### Associations between procedure characteristics and SSL detection rates (SDR)

**Fig. 2:**
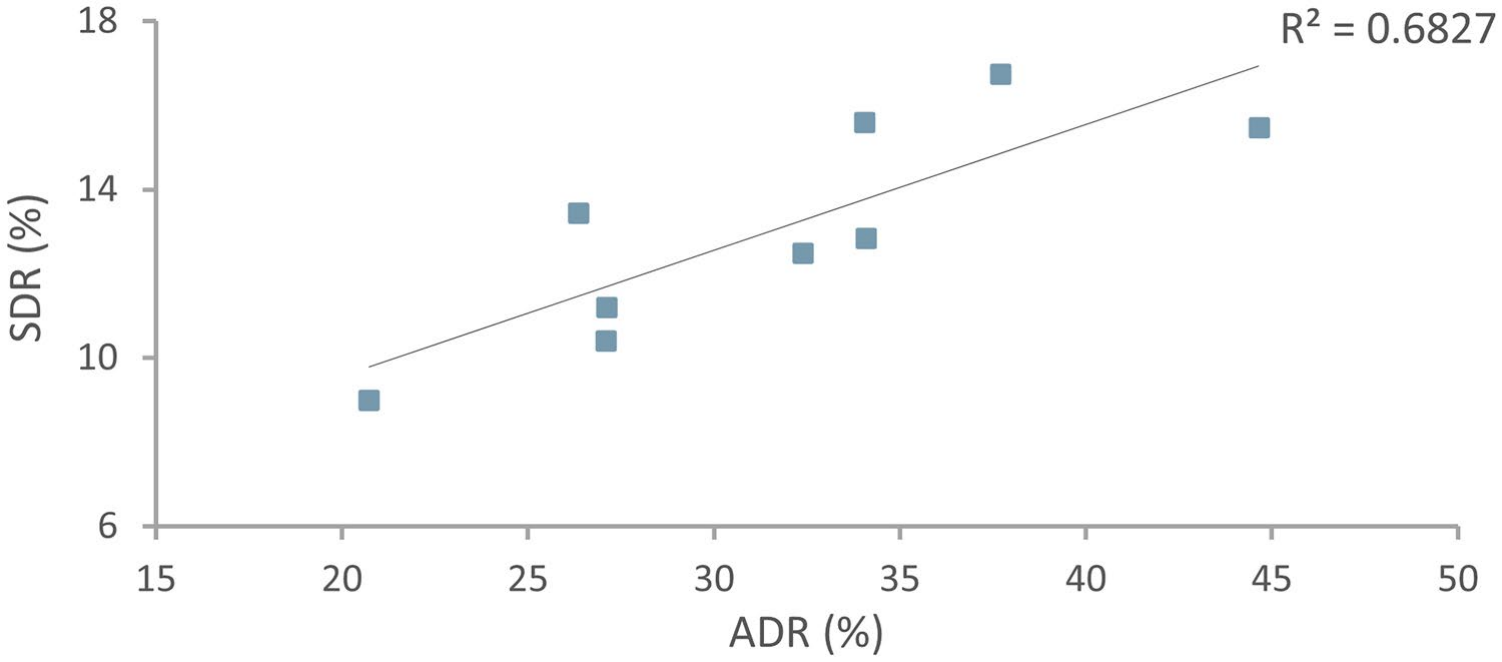
Individual sessile serrated lesion detection rate (SDR) vs. conventional adenoma detection rate (ADR) of colonoscopists performing more than 100 colonoscopies (*n* = 9)

Table 4 summarises the associations between procedure characteristics and SSL detection. SDRs ranged between 9 and 16.8% among colonoscopists. SSL detection was higher in patients with synchronous adenomas and HPs. Furthermore, SDR was significantly correlated with individual endoscopist ADR (*r* = 0.83, *n* = 9, *p* = 0.002). The relationship between individual SDR and ADR is shown in Fig. 2. There was no association between SSL detection and the presence of diverticular disease.

**Table 4:** Associations between individual procedural characteristics and SSL detection

**Procedural factor**		Total*	**SSLs**		***p*** **value**
			***Yes (n = 288)***	***No (n = 1800)***	
**Conventional adenomas**					**0.004**
* No*		1387 (66.4%)	**170 (12.3%)**	1217 (87.7%)	
* Yes*		701 (33.6%)	**118 (16.8%)**	583 (83.2%)	
**Hyperplastic polyps**					** < 0.001**
* No*		1536 (73.6%)	**184 (12.0%)**	1352 (88.0%)	
* Yes*		551 (26.4%)	**104 (18.9%)**	447 (81.1%)	
**Diverticular disease**					
< *50 years*					0.13
* No*		734 (86.8%)	87 (11.9%)	647 (88.1%)	
* Yes*		112 (13.2%)	19 (17.0%)	93 (83.0%)	
≥ *50 years*					0.54
* No*		631 (55.3%)	111 (15.2%)	620 (84.8%)	
* Yes*		510 (44.7%)	71 (13.9%)	439 (86.1%)	

*SSL* sessile serrated lesion

*Totals less than the overall number of colonoscopies (2097) occur where data is missing for the respective variables.

#### Associations between procedure characteristics and adenoma detection

Adenoma detection ranged between 21.5 and 45% among colonoscopists. Coincident colonoscopy findings associated with adenoma detection were synchronous SSL detection (OR 1.45. 95% CI 1.12–1.87) and the presence of diverticular disease (OR 1.78, 95% CI 1.47–2.16).

### Multivariable analysis

#### Patient cohort and procedure factors associated with SSL detection and CSSP detection

Factors included in multivariable logistic regression analysis for SSL detection were patient age (categorical < versus ≥ 50 years), sex, BMI (categorical < versus ≥ 25 kg/m^2^), synchronous adenoma detection, and bowel preparation. SSL detection was associated with female sex (aOR 1.48, 95% CI 1.15–1.91), synchronous adenoma detection (aOR 1.36, 95% CI 1.04–1.78), and BMI ≥ 25 kg/m^2^ (aOR 1.34, 95% CI 1.02–1.77). With HPs included in the model, factors associated with SSL detection were female sex (aOR 1.48, 95% CI 1.15–1.91), synchronous adenoma detection (aOR 1.37, 95% CI 1.04–1.79), and synchronous HP detection (aOR 1.64, 95% CI 1.26–2.15). For CSSP detection, multivariable logistic regression included SSL detection, BMI, synchronous adenoma detection, and bowel preparation. Factors associated with CSSP detection were female sex (aOR 1.40, 95% CI 1.11–1.75) and synchronous adenoma detection (aOR 1.43, 95% CI 1.12–1.83).

#### Patient cohort and procedure factors associated with adenoma detection

Factors included in multivariable logistic regression analysis for adenoma detection were patient age (categorical < versus ≥ 50 years), BMI (categorical < versus ≥ 25 kg/m^2^), a positive FIT, synchronous SSL detection, diverticular disease, and bowel preparation. Adenoma detection was associated with age ≥ 50 (aOR 2.24, 95% CI 1.66–3.00) and synchronous SSL detection (aOR 1.79, 95% CI 1.30–2.48).

## Discussion

In this outpatient colonoscopy population that included adults aged between 18 and 75 years and various indications, we found a SDR of 13.8% overall and 12.5% in patients < 50 years. The rate of SSL detection was significantly higher in females compared to males, with this sex difference being most significant in patients < 50 years of age. Female sex, synchronous adenoma detection, and BMI > 25 kg/m^2^ significantly increased the odds of SSL detection in multivariable analysis. None of the three indication categories (blood loss, non-bleeding gastrointestinal symptoms, or surveillance/screening) were associated with SSL detection. The majority (64%) of SSLs were found in the proximal colon. There was no significant association between SSL detection and a diagnosis of diverticular disease.

### Risk factors for SSL and adenoma detection

#### Age and sex

Our findings suggest females under 50 years may be at increased risk for SSL detection. This was independent of the quality of bowel preparation, which would be expected to influence the yield of polyp detection. CRC screening guidelines in Australia are currently not sex-specific, with two-yearly FIT recommended for all screening average-risk individuals aged 50–74 years, and colonoscopy performed following a positive result [22]. The association between sex and SSL detection, however, is unclear. Some studies identify equal prevalence among males and females, whereas others find either males or females to have a higher prevalence. This variability in sex association is observed regardless of geographical region. Australian studies have reported either no association with sex or association with female sex [16, 23]. On the other hand, European studies report either no association or association with the male sex [24, 25]. Studies derived from the US populations generally report no association with sex, whereas South American studies have reported an association with female sex [14, 26–28].

There are several potential reasons why our study, and others, exhibit considerable variability in SSL sex associations and SSL prevalence. Firstly, the dates of publication of the above studies range from 2008 to 2019, suggesting that different histopathological criteria have been used to identify SSLs [14, 16, 23–28]. The most recent SSL histopathological criteria are more sensitive than those used previously, possibly contributing to our relatively high-observed prevalence. Moreover, there is high variability in both endoscopist and histopathologist detection/diagnosis of SSLs, with recorded endoscopist detection rates of 0.6–20.1% and pathologist classification rates of 0.5–12.0% [4, 17, 29]. In addition to changing pathological criteria, it is likely the above variation is due to differences in endoscopist/histopathologist skills since both colonoscopy and histopathological analysis are subjective techniques. The histopathological assessment of all polyps in our study was provided by specialist histopathologists from the state specialist tertiary pathology service. Finally, different studies may have involved patient cohorts with inherently different characteristics and susceptibilities to SSL risk due to unknown confounding variables. The reliability and generalisability of previous research are therefore unclear. Contemporary, large-scale studies are needed to provide more robust evidence regarding risk factors for SSL detection.

Existing data is also inconsistent regarding age as a risk factor for SSLs, with some studies reporting advanced age as a risk factor and other studies finding no correlation. Reasons for this variability are similar to those regarding sex. A recent Australian study by Kim et al. [3] identified rising CRC rates in patients aged < 50 years, particularly in males. Furthermore, another recent paper by Sehgal et al. [30] concluded that colonoscopy at ages 45–49 was associated with a considerable decrease in CRC incidence in both males and females. Lash et al. [27] reported that dysplasia and carcinoma progression were disproportionately higher in female patients with SSLs compared to male patients. In view of the above findings, and the emergence of new diagnostic criteria for SSLs, further data is needed to determine the adequacy of current screening guidelines and benchmarks for SSL detection.

In contrast to SSLs, we found that adenoma detection was significantly higher in patients ≥ 50 than < 50 years, with no significant difference between males and females. We also noted that while SSLs and CSSPs were more prevalent in females up to age 59 years, no sex difference was seen with adenomas. While our findings concur with previous studies in terms of age associations, previous literature has shown higher adenoma detection in males compared to females [31–33]. With regard to baseline characteristics between our cohort’s males and females, mean BMI and age did not significantly differ. There may be other confounding risk factors creating a bias that was not captured in our data and contributed to a relatively high prevalence of adenomas in females. These include smoking, ethnicity, other past medical histories, particular incidental findings, family history of cancer, and diet.

#### Other risk factors

Other risk factors for SSL detection exhibit varied significance in the literature, with tobacco, alcohol, and the white race being among the most consistently identified [4, 18]. Many of these risk factors could not be examined in our study given their inconsistent reporting. BMI was associated with SSL detection in univariate and multivariable analysis excluding HPs. The significance of such findings, however, remains largely unclear, as while studies show associations between serrated lesion detection or adenoma detection and BMI [34, 35], few studies focus specifically on SSLs. Whether or not sex hormones and adiposity contribute to SSL risk in young women remains unclear. Such a link between sex hormones and early colorectal carcinogenesis has been suggested in a previous study by Hang et al.; however, specific associations for SSLs were not explored [36]. Although the diverticular disease was not associated with SSL detection in our study, there was an association between diverticular disease and adenomas in our univariate analysis and also in previous studies and meta-analyses [2, 37, 38]. Further studies using contemporary diagnostic criteria for SSLs may elucidate associations between SSLs and the risk factors above. Finally, synchronous adenomas were associated with SSL detection in both univariate and multivariate analyses. This is consistent with previous studies suggesting SSLs increase the risk of synchronous and metachronous neoplasia [39, 40].

#### Colonoscopy indication

Although we found that no colonoscopy indication was associated with SSL detection, this is a complex variable to study. Patients often had numerous indications, making the association of individual indications with SSL detection difficult to analyse. Furthermore, because indications were grouped into three different categories, conclusions can only be made about indication categories as a whole. We also found synchronous adenoma detection was associated with SSL detection. Therefore, if a particular indication was associated with adenoma detection, this would likely associate with SSL detection regardless of whether it was an independent covariate. Significantly, FIT status was not associated with SSL detection, consistent with the fact that SSLs rarely bleed [4, 16]. This means that while some studies support earlier initiation of FIT for increasing rates of CRC in average-risk patients < 50 years, such an approach may provide inadequate protection for females < 50 years who may be more susceptible to SSLs [3]. Whether there are specific characteristics that would justify screening with colonoscopy in females < 50 years remains to be explored.

### SSL prevalence and current detection benchmarks

The standard quality indicator for colonoscopist performance in screening colonoscopy is the ADR, i.e., the proportion of colonoscopies performed in which at least one adenoma is detected and removed [41, 42]. The currently accepted benchmark ADR for endoscopists is ≥ 25% [43], which has been used by some studies to derive benchmarks for SSL detection. The correlation between ADR and SDR, however, remains uncertain, with certain studies showing moderate to high correlation (as reflected in our findings) and others suggesting otherwise [18, 44, 45]. The Cancer Council Australia (CCA) recommends a serrated lesion detection rate of > 10% [46]. Nevertheless, this benchmark includes HPs, which are more prevalent than SSLs but are not considered premalignant [4]. As for SSL-specific benchmarks, GESA proposes a 4% detection threshold for SSLs [19]. Such a rate, however, may be inadequate considering the rising prevalence rates of SSLs and our detection rate of 13.8%. Our findings, therefore, support the revision of benchmarks for SSL detection as additional data emerges. Previous studies indicate that the mortality benefit of colonoscopy screening has been limited to distal CRC [47–49]. This suggests that enhancing the detection of SSLs, which are mostly proximal in location [4], will significantly improve the effectiveness of colonoscopy screening.

### Significance of SSLs in patients under 50 years

The significance of our finding of higher rates of SSLs in younger females is uncertain, particularly given it is a novel finding that is not described extensively in the literature. A study from the USA provides reassurance that proximal SSLs have a relatively low likelihood of progressing to cancer in young people [50]. This is attributed to the fact that most young-onset CRC (onset < 50 years) is distal and exhibits different molecular characteristics than SSLs [51]. A more recent study by Hamoudah et al., however, suggests an increased risk of metachronous advanced colorectal neoplasia when small, serrated lesions and adenomas coexist [52]. This means that when accounting for other factors like synchronous adenoma detection and variable SSL prevalence and sex distribution, the association between SSLs and young-onset CRC becomes less clear. Our study found that SSLs frequently coexist with adenomas−a major precursor to young-onset CRC [50]. Furthermore, when considering Australian data, we find that 24% of cases of young-onset CRC diagnosed between 2001 and 2008 were proximal in location, meaning some could have been contributed to by SSLs or risk factors shared with adenomas [53]. While some studies have demonstrated a high prevalence of SSLs in a general colonoscopy population [54], few studies have investigated the significance of a potentially higher prevalence of SSLs in patients under 50 years. The fact that 16.8% of females under 50 years in our study had SSLs may warrant further consideration and examination of how such detection rates may correlate with young-onset CRC. Although a study by Lash et al. [27] demonstrates slow rates of SSL progression to dysplasia and carcinoma, another study by Oono et al. suggests that such progression may also occur rapidly [55]. Therefore, while most SSLs in our study occurred without dysplasia, the natural history of SSLs and the risk they pose to females under 50 years with potential susceptibility to SSLs remains unclear. Finally, although young-onset CRC tends to occur distally whereas SSLs tend to occur proximally, a considerable proportion of SSLs in our study were distal (> 30%), including in patients < 50 years. Further studies using prospective, multicentre, and contemporary histopathological data are required to elucidate the significance of these findings.

### Strengths and limitations

Our study has several strengths, including a large and reasonably well-characterised population and reliance on histopathologic descriptions of polyps. We observed a high photo-documented caecal intubation rate and reasonably high-quality bowel preparation. Additionally, our data involved specialist colonoscopists without outlier SDRs, which adds generalisability to our findings and reduces the influence of inter-endoscopist variability. We also provide data that is likely to reflect contemporary diagnostic criteria for serrated lesions. As for limitations, the retrospective design of our study means it is limited by some missing or inconsistently reported data (for example smoking and ethnicity) and unknown biases. It is also unclear what proportion of patients had a prior colonoscopy. However, less than 20% of colonoscopies were performed for surveillance (the majority, approximately 15%, being for previous polyp detection), suggesting that most procedures were not for polyp surveillance.

Data regarding IBD in our study was limited as IBD patients comprised < 1% of our patient cohort. Nevertheless, associations between IBD and SSLs remain unclear [56]. In terms of colonoscopy characteristics, withdrawal times were not consistently documented, which leads to uncertainty regarding adherence to the recommended > 6-min withdrawal times [57]. Similarly, chromoendoscopy use was poorly documented, suggesting the technique was rarely used in this cohort. Furthermore, bowel preparation was recorded based on colonoscopist impression (graded as excellent, adequate, fair, inadequate, or poor) rather than validated scoring systems such as the Boston Bowel Prep Score. [58] Regarding histopathology, there is likely to be variability in our study in terms of diagnostic criteria for SSLs given that multiple pathologists (15) were involved in polyp diagnosis. It was, therefore, difficult to ascertain what proportion of pathologists utilised the 5th edition versus the 4th edition of the WHO criteria for SSL diagnosis. However, all pathologists worked in a tertiary teaching hospital capacity. As newer criteria are more sensitive for SSL diagnosis, our findings likely underestimate rather than overestimate the true prevalence of SSLs. Addressing the above limitations requires further, high-quality, large prospective studies.

## Conclusion

Our study identified an SSL prevalence of 13.8% in an outpatient colonoscopy population, with females < 50 years being at considerable risk. Other associated factors included the presence of synchronous adenomas, which indicates a potential subsequent risk of advanced colorectal neoplasia. These findings suggest that current SDR benchmarks and CRC screening guidelines may be inadequate, particularly if females < 50 years are at increased risk of CRC via the serrated-neoplasia pathway. Whether or not differences in sex-hormone levels with age play a role in the increased susceptibility of young females to SSLs remains to be elucidated. Further studies are required to resolve the significance of our findings.
